# Organ dose and total effective dose of whole-body CT in multiple myeloma patients

**DOI:** 10.1007/s00256-019-03292-z

**Published:** 2019-10-15

**Authors:** Robert Hemke, Kai Yang, Jad Husseini, Miriam A. Bredella, F. Joseph Simeone

**Affiliations:** 1 Division of Musculoskeletal Imaging and Intervention, Department of Radiology, Massachusetts General Hospital, Harvard Medical School, Boston, MA 02114 USA; 2 Department of Radiology and Nuclear Medicine, Amsterdam University Medical Centers, Location Academic Medical Center, Amsterdam Movement Sciences, Meibergdreef 9, 1105 AZ Amsterdam, The Netherlands; 3 Division of Diagnostic Imaging Physics, Department of Radiology, Massachusetts General Hospital, Harvard Medical School, Boston, MA 02114 USA

**Keywords:** Multiple myeloma, Whole-body low-dose CT, Effective dose, Organ dose, Skeletal survey

## Abstract

**Objective:**

To evaluate organ dose and total effective dose of whole-body low-dose CT (WBLDCT) performed on different CT-scanner models in patients with multiple myeloma (MM) and to compare it to the effective dose of radiographic skeletal survey and representative diagnostic CTs.

**Material and methods:**

We retrospectively analyzed data from 228 patients (47.4% females, mean age 67.9 ± 10.4 years, mean weight 81.8 ± 22.4 kg) who underwent WBLDCT for the work-up or surveillance of MM. Patients were scanned using one of six multi-detector CT-scanners. Organ doses and total effective doses per scan were calculated using a commercially available dose-management platform (Radimetrics, Bayer Healthcare, Leverkusen, Germany). The median effective dose was compared to radiographic skeletal survey and representative diagnostic CTs.

**Results:**

The mean effective dose of our WBLDCT-protocol was 4.82 mSv. A significantly higher effective dose was observed in females compared to males (4.95 vs. 4.70 mSv, *P* = 0.002). Mean organ dose ranged from 3.72 mSv (esophagus) to 13.09 mSv (skeleton). Mean effective dose varied amongst different CT-scanners (range 4.34–8.37 mSv). The median effective dose of WBLDCT was more than twice the dose of a skeletal survey (4.82 vs. 2.04 mSv), 23% higher than a diagnostic contrast-enhanced chest CT (3.9 mSv), 46% lower than a diagnostic contrast-enhanced abdomen/pelvis CT (9.0 mSv), and 45% lower than a lumbar spine CT (8.7 mSv).

**Conclusions:**

WBLDCT in MM has a higher effective dose than a radiographic skeletal survey, but a lower effective dose than diagnostic CTs of lumbar spine, abdomen and pelvis. This underlines the broad applicability of WBLDCT in the management of MM patients.

## Introduction

Multiple myeloma (MM) is the second most common hematological malignancy with an incidence that has risen over the past decades [1, 2]. It is characterized by the abnormal proliferation of monoclonal plasma cells in the bone marrow, which can lead to replacement of normal myelopoiesis, bone destruction and bone marrow failure [3]. Diagnosis of active MM as defined by the International Myeloma Working Group (IMWG) originally relied on histological confirmation of bone marrow plasma cell infiltration with clonal bone marrow plasma cells ≥10% (or extramedullary plasmacytoma) and one of the following features: hypercalcemia, renal insufficiency, anemia and bone lesions [4]. According to the IMWG, the presence of lytic bone lesions is considered one of the criteria to define active disease that requires treatment, even in the absence of clinical symptoms [4]. Consequently, imaging plays an important role in staging and in the follow-up of the disease [3, 5], as well as for the initial work-up of patients with smoldering MM [6].

Skeletal survey using approximately 20 regional radiographs has been used to determine osteolytic lesions or pathological fractures from MM. However, the value of conventional radiographs in detecting osteolytic lesions is limited since at least 30% of trabecular bone must be destroyed to be visible by radiograph [7]. Cross-sectional imaging techniques such as computed tomography (CT), magnetic resonance imaging (MRI) and integrated positron emission tomography/CT (PET/CT), have proven to be more accurate in detecting osteolytic and marrow replacing lesions as compared to skeletal surveys using radiographs [8–14]. As a result, more accurate diagnosis, staging and follow-up of MM is possible [8, 9].

Whole body low-dose CT (WBLDCT) was found to be the most sensitive modality for detecting small osteolytic lesions (<5 mm) [15]. Moreover, WBLDCT provides information for disease monitoring and detection of incidental findings in MM patients, underlining its importance in the management of this patient group [16]. Although advances in CT technology enable low-dose whole-body evaluation, CT is limited by ionizing radiation exposure. While some literature on WBLDCT in MM report radiation dose information [17], current literature lacks dedicated data in larger patient cohorts on the organ dose exposure and effective radiation exposure in the clinical practice of WBLDCT’s in MM patients. Therefore, the objective of our study was primarily to evaluate the organ-specific-radiation dose levels and total effective dose of WBLDCT in a MM patient cohort and to compare them to calculated effective doses of a skeletal survey and representative diagnostic CT scans. We hypothesized that the effective dose of WBLDCT would be lower than that of diagnostic CTs and higher than that of a skeletal survey.

## Materials and methods

Our study was IRB-approved and complied with HIPAA guidelines with exemption status for individual informed consent. ﻿A retrospective search was performed of all WBLDCT’s acquired from July 2017 to February 2019.

### Whole-body low-dose CT protocols

CT examinations were performed without administration of intravenous or oral contrast medium on one of our six multidetector CT scanners (SOMATOM Force, SOMATOM Definition Flash, SOMATOM Definition Edge; Siemens, Forchheim, Germany; GE Discovery, GE Revolution; VCT, Boston, MA, USA; IQon Spectral, Philips, Best, the Netherlands). Patients were scanned in supine position, with head first and the arms straight beside the body and hands placed over the upper thighs. The field of view was adapted to the patient’s circumference.

WBLDCT imaging parameters per used device are depicted in Table 1.

**Table 1:** Whole-body CT imaging parameters per device

Device	No. of slices per rotation	Tube voltage (kVp)	Tube current modulation parameters	Collimation width	Rotation time (s)	Pitch
Revolution CT, GE	256	120	20 (noise index)	80 mm	0.5	0.992
Discovery CT750, GE	64	120	20 (noise index)	40 mm	0.5	0.984
Definition Edge, Siemens	128	120	70 (quality reference mAs)	38.4 mm	0.5	1
Definition Flash, Siemens	128	120	70 (quality reference mAs)	38.4 mm	0.5	1
SOMATOM Force, Siemens	192	120	70 (quality reference mAs)	57.6 mm	0.5	1
IQon, Philips	128	120	12 (dose right index)	40 mm	0.5	1.171

### Radiation dose assessment of whole-body low-dose CT

We assessed the radiation dose in terms of organ dose and whole-body effective dose using a commercial dose monitoring and tracking software platform (Radimetrics, Bayer Healthcare, Leverkusen, Germany). Based on anthropomorphic phantoms and Monte Carlo simulations, this software extracts volume computed tomography dose index (CTDI_vol_), dose-length product (DLP) from each scan and provides organ dose values (mSv) and total effective dose (mSv) per CT scan according to the weighting factors published in the International Commission on Radiation Protection 103 report [18]. Regarding the organ dose, values for the following organs were recorded: adrenals, brain, colon, esophagus, eye lenses, gall bladder, heart, kidneys, liver, lungs, muscle, pancreas, red marrow, salivary glands, skeleton, skin, small intestine, spleen, stomach, thyroid, thymus and urinary bladder. Furthermore, the organ dose of breasts, ovaries and the uterus were recorded in female subjects as well as the testis dose in males.

### Radiation dose calculation of a skeletal survey and representative diagnostic CT scans

At our institution, the radiographic skeletal survey includes the following 22 different images; skull (two directions), cervical spine (two directions), thoracic spine (two directions), lumbar spine (two directions), chest (PA), pelvis (AP), humerus (AP, bilateral), forearm (AP, bilateral), hip (AP, bilateral), femur (AP, bilateral), knee (AP, bilateral), and tibia/fibula (AP, bilateral). The median effective dose (mSv) per exam was calculated using our institution’s median data of dose-area-product (DAP) and conversion factors as described by Wall et al. [19]. The sum of the effective dose per exam resulted in the total effective dose of the skeletal survey used in our institution.

In order to put the total effective dose of the MM WBLDCT protocols into perspective, the median effective dose of the following commonly used diagnostic CT protocols without the use of intravenous contrast was calculated with DLP, using our institution’s median data from 1/1/2017 to 9/14/2018 from the ACR dose index registry (DIR) and conversion factors from AAPM report 96 [20]—brain, cervical spine, thoracic spine, lumbar spine, chest (low dose/screening protocol), and abdomen/pelvis. Similarly, the median effective dose of diagnostic chest and abdomen/pelvis CT protocols with the use of intravenous contrast was calculated.

### Statistical analysis

Descriptive statistics were reported in terms of percentages, means, standard deviations (SD), medians and interquartile ranges (IQR). The independent samples T test was used to analyze differences in effective dose and organ dose between male and female patients. The test assumed a two-tailed probability and a *P*-value of less than 0.05 indicated a significant difference.

## Results

In this retrospective study, we analyzed data of 228 patients—108/228 (47.4%) females, mean age 67.9 (SD 10.4) years, mean weight 81.8 (SD 22.4) kg. An overview of the total organ dose and differences between organ dose in males and females is depicted in Table 2. In the total group, the mean organ dose ranged from 3.72 (SD 1.01) mSv for the esophagus up to 13.09 (SD 2.84) mSv for the skeleton. As shown in Table 2, differences were observed between males and females regarding the mean organ dose of the thyroid (10.25 (SD 1.55) mSv vs. 8.27 (SD 1.42) mSv, *P* < 0.001, respectively), the esophagus (3.58 (SD 0.52) mSv vs. 3.75 (SD 0.58) mSv, *P* = 0.019, respectively), and the colon (4.06 (SD 0.45) mSv vs. 4.21 (SD 0.63) mSv, *P* < 0.001, respectively).

**Table 2:** Organ dose (mSv) ^a^. *Italics* shows differences observed between males and females regarding the mean organ dose of thyroid, esophagus and colon

	Total	Male	Female	*P* value^b^
	*n* = 228 (100%)	*n* = 120 (52.6%)	*n* = 108 (47.4%)	
Head and neck				
Brain	7.42 (1.57)	7.44 (1.08)	7.26 (1.38)	0.267
Eye lenses	9.75 (2.14)	9.85 (1.59)	9.46 (1.84)	0.089
Salivary glands	7.42 (1.57)	7.44 (1.08)	7.26 (1.38)	0.267
Thyroid	9.41 (2.35)	10.25 (1.55)	8.27 (1.42)	*<0.001*
Chest				
Breast	NA	NA	5.55 (0.86)	NA
Heart	4.78 (1.34)	4.71 (0.72)	4.69 (0.70)	0.852
Lungs	5.45 (1.45)	5.40 (0.83)	5.33 (0.79)	0.500
Gastro-intestinal system				
Esophagus	3.72 (1.01)	3.58 (0.52)	3.75 (0.58)	*0.019*
Stomach	4.81 (1.28)	4.71 (0.56)	4.77 (0.72)	0.489
Small intestine	4.06 (1.12)	3.95 (0.47)	4.04 (0.61)	0.216
Colon	4.20 (1.13)	4.06 (0.45)	4.21 (0.63)	*0.048*
Abdominal organs				
Liver	4.76 (1.25)	4.65 (0.56)	4.74 (0.71)	0.249
Gall bladder	4.28 (1.18)	4.20 (0.53)	4.22 (0.68)	0.761
Pancreas	3.94 (1.08)	3.82 (0.47)	3.93 (0.58)	0.117
Spleen	4.70 (1.21)	4.57 (0.54)	4.69 (0.70)	0.140
Adrenal	4.17 (1.17)	4.10 (0.52)	4.10 (0.67)	0.982
Kidneys	5.02 (1.34)	4.96 (0.60)	4.92 (0.73)	0.669
Urinary bladder	4.68 (1.32)	4.64 (0.59)	4.58 (0.67)	0.464
Reproductive system				
Ovaries	NA	NA	3.79 (0.59)	NA
Uterus	NA	NA	3.97 (0.61)	NA
Testicles	NA	8.48 (2.48)	NA	NA
Musculoskeletal system and skin				
Skeleton	13.09 (2.84)	12.72 (1.70)	13.21 (2.09)	0.058
Red marrow	4.30 (1.02)	4.19 (0.48)	4.30 (0.60)	0.128
Muscle	5.71 (1.30)	5.62 (0.74)	5.66 (0.85)	0.677
Skin	6.72 (1.43)	6.74 (0.95)	6.56 (1.04)	0.169

^a^Values are means (SD)

^b^*P*-values indicate differences between groups. The independent samples T-test was used to analyze differences between groups

*NA* not applicable/available

The mean total effective dose of our WBLDCT protocol was 4.82 (SD 0.62) mSv. A significant higher effective dose was observed in females compared to males; 4.95 (SD 0.70) mSv vs. 4.70 (SD 0.51) mSv, respectively, *P* = 0.002.

The majority of the WBLDCT scans were obtained using the Revolution CT, GE Medical Systems (198 [86.8%)], followed by the SOMATOM Definition Edge, Siemens [12 (5.3%)], SOMATOM Definition Flash, Siemens [7 (3.1%)], IQon - Spectral CT, Philips [7 (3.1%)], Discovery CT750 HD, GE Medical Systems [3 (1.3%)), and the SOMATOM Force, Siemens [1 (0.4%)]. The effective dose, CTDI_vol_ and DLP per used device are depicted in Table 3. The mean effective dose ranged from 4.34 mSv (SOMATOM Force, Siemens) up to 8.37 mSv (Discovery CT750 HD, GE Medical Systems).

**Table 3:** Effective dose, CTDI_vol_ and DLP between devices^a^

	Revolution CT	Discovery CT750	Definition Edge	Definition Flash	Force	IQon
	GE	GE	Siemens	Siemens	Siemens	Philips
	*n* = 198 (86.8%)	*n* = 3 (1.3%)	*n* = 12 (5.3%)	*n* = 7 (3.1%)	*n* = 1 (0.4%)	*n* = 7 (3.1%)
Effective dose (mSv)	4.73 (4.52–4.98)	8.37 (7.32-NA)	4.47 (4.24–4.74)	5.09 (4.61–6.10)	4.34 (NA)	5.20 (4.55–5.47)
CTDI_vol_ (mGy)	4.10 (3.61–4.41)	6.05 (5.40-NA)	3.91 (3.41–4.61)	5.30 (5.19–5.67)	3.61 (NA)	4.20 (4.00–4.69)
DLP (mGy•cm)	561 (486–614)	784 (687-NA)	510 (463–669)	711 (664–719)	515 (NA)	569 (528–760)

^a^Values are medians (IQR)

*DLP* dose length product, *CTDI*_*vol*_ volume computed tomography dose index, *NA* not applicable/available

Table 4 shows the calculated effective doses of a skeletal survey and representative commonly used diagnostic CT scans at our institution. The median effective dose of our skeletal survey (including 22 radiographs) was 2.04 mSv. The calculated median effective dose of representative diagnostic CT scans at our institution ranged from 1.2 mSv for a low-dose chest CT up to 9.0 mSv for a contrast-enhanced CT of the abdomen/pelvis.

**Table 4:** Effective doses of a skeletal survey and commonly used diagnostic CT scans at Massachusetts General Hospital (MGH)

Exam	IV contrast^a^	Effective dose (mSv)	
		MGH Median	ACR DIR Median
Skeletal survey (22 images)	NA	2.04	NA
CT Brain	No	1.6	1.8
CT Cervical spine	No	2.1	2.7
CT Thoracic spine	No	NA	13.1
CT Lumbar spine	No	8.7	11.2
CT Chest low dose protocol	No	1.2	1.2
CT Chest	Yes	3.9	6.0
CT Abdomen/pelvis	Yes	9.0	10.2
CT Abdomen/pelvis	No	8.4	10.0

^a^CT protocol with the use of intravenous contrast

*NA* not applicable/available

## Discussion

Our study showed that WBLDCT in MM patients has a higher effective dose than that of a radiographic skeletal survey, but a lower effective dose than that of diagnostic CTs of the lumbar spine, abdomen and pelvis. Within the past decades the survival of MM patients has improved because of the availability of novel treatment options [21–23]. The presence of these treatment options had underlined the importance of early diagnosis and accurate staging [8]. According to the most recent IMWG diagnostic criteria, lesions measuring 5 mm on CT or MRI are now included in the diagnostic criteria of MM [4], and it is of clinical importance to detect bone involvement in MM and its precursors. Moreover, in MM patients, bone involvement is an important cause of morbidity and mortality and a key indicator of prognosis [4].

Although skeletal surveys using radiographs have been traditionally used to assess for bone involvement in MM patients, its limitations are well known and have been documented previously [8]. Therefore, more sensitive imaging techniques for the diagnosis and follow-up of MM have been described, such as WBLDCT, MRI and PET/CT. Whole-body MRI and 18F-fluorodeoxyglucose-PET/CT have been described to be more accurate in detecting bone lesions in MM as compared to skeletal surveys [8–14], and whole-body MRI have been found to detect significantly more MM bone involvement compared to PET/CT [12]. Whole-body MRI and PET/CT are, however, limited in their availability and are relatively costly. Additionally, FDG-PET/CT results in a relatively high radiation dose to the patient.

The widespread availability of multi-detector CT scanners has contributed to the increased use of WBLDCT for the diagnosis and follow-up of MM. The total effective dose of the WBLDCT in our study is comparable to those reported in recent literature [24–27]. Moreover, our measured organ specific dose is comparable to the organ dose described by Kröpil et al. [28]. In our study, the effective dose of WBLDCT was, as expected, higher than that of a radiographic skeletal survey; however, the total effective dose of WBLDCT proved to be lower than that of commonly used diagnostic CTs of the lumbar spine, abdomen and pelvis. Moreover, WBLDCT has been shown to provide important information for disease monitoring and detection of incidental findings [16], thereby improving the management of MM patients.

In recent literature, WBLDCT protocols with estimated effective doses comparable to or lower than radiographic skeletal surveys have been described, using for instance hybrid iterative reconstruction techniques [10] or spectral shaping [24]. Although these studies show the potential for further decreasing radiation exposure of WBLDCT in MM, the radiation doses in our study reflect those that result from current daily practice.

Our study was limited by the retrospective study design. Secondly, over 85% of our WBLDCT scans were performed using one scanner, the Revolution CT, GE Medical Systems. This resulted in a relative underrepresentation of the other systems used. Strengths of our study include the large number of patients scanned using a uniform clinical protocol and the detailed assessment of radiation dose.

In conclusion, WBLDCT in MM has a higher effective dose than a radiographic skeletal survey, but a lower effective dose than diagnostic CTs of the lumbar spine, abdomen and pelvis. This underlines the broad applicability of WBLDCT in the management of MM patients. The additional diagnostic value of WBLDCT in the management of MM patients outweighs the relatively limited additional radiation dose as compared to a radiographic skeletal survey.
